# Myelinated retinal nerve fiber layer: an extensive presentation

**DOI:** 10.11604/pamj.2022.42.51.34665

**Published:** 2022-05-18

**Authors:** Zeineb Kallel, Malek Kharrat

**Affiliations:** 1 Ophtalmology Department, Hospital of Mohamed Taher Maamouri, Nabeul, Tunisia

**Keywords:** Myelinated retinal fibers, amblyopia, strabismus

## Image in medicine

Myelinated retinal nerve fiber layer is rare. It is estimated to occur in 0.5% to 1% of the population and is often detected on routine examination without symptoms. It consists of white or gray-white well-demarcated patches on the outermost surface of the retina that obscure the underlying retinal vessels. It can be associated with axial myopia, amblyopia, and strabismus. A 12-year-old girl with normal development presented with ocular deviation of the right eye. There was no past medical or ocular history. Automatic refraction found in the right eye -9 Pupillary Distance (PD) and -1 PD in the left eye. The best visual acuity was 20/600 in the right eye and 20/20 in the left one. Slit lamp examination of the right eye (A) revealed two vast whitish zones extending from the papilla towards the temporal sector sparing the posterior pole. These lesions covered the temporal vessels with fuzzy and scalloped boundaries. Fundus examination of the left eye was without abnormalities (B). Scan of Spectral Domain Optical Coherence Tomography (SD-OCT) showed isolated hyper reflectivity of the fiber optic layer, an abnormality of the vitreoretinal interface with posterior shadow cone effect (C).

**Figure 1:**
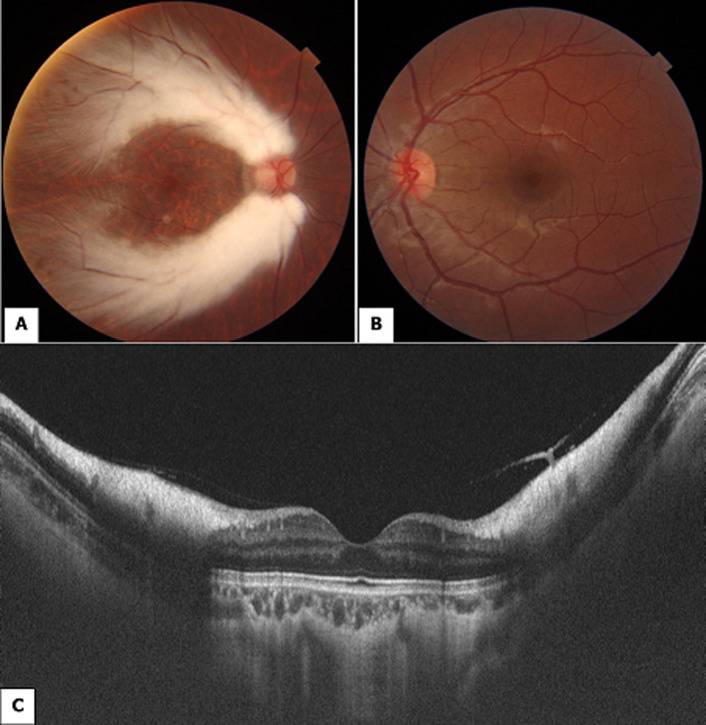
fundus examination of both eyes and SD-OCT scan: A (Right Eye (RE)): two vast whitish zones extending from the papilla towards the temporal sector sparing the posterior pole; B (Left Eye (LE)): without abnormalities; C (SD-OCT): hyper reflectivity of the retinal nerve fiber layer with posterior shadow cone effect.

